# Insights into the mechanisms of microbiome and metabolome changes mediated by understory interplanting mode in *Polygonatum sibiricum*

**DOI:** 10.3389/fmicb.2023.1232846

**Published:** 2023-08-10

**Authors:** Yue Wang, Jin Zhang, Jiabo Sun, Guoqing Li, Qian Wang, Yanxia Zhao, Changjian Ma, Jinlong Han

**Affiliations:** ^1^Shandong Academy of Agricultural Sciences, Jinan, China; ^2^Tai'an Taishan Forestry Research Institute, Tai’an, China; ^3^Tai'an Academy of Agricultural Sciences, Tai’an, China; ^4^Shandong Taishang Huangjing Biotechnology Co., Ltd., Tai’an, China

**Keywords:** *Polygonatum sibiricum*, understory interplanting mode, metabolome, microbiome, endophyte, rhizosphere soil

## Abstract

**Background:**

*Polygonatum sibiricum* is an understory economic plant, and its dried rhizome is a traditional Chinese medicine. The purpose of this study was to connect the quality improvement of the understory plant *P. sibiricum* with specific microorganisms.

**Methods:**

Amplicon and metabolome sequencing were conducted for *P. sibiricum* interplanted under three types of trees and in the field, and the relationship between the microbiome and secondary metabolism was explored.

**Results:**

Principal component analysis (PCA) divided field cultivated and understory interplanted groups into two classes. A total of 95 different metabolites were found, with four expression patterns. The alpha diversity of rhizosphere bacteria and endosphere fungi in the understory interplanted group was significantly higher than that in the farmland cultivated group. There were 276 different rhizosphere microorganism genera among the four groups; however, only 33 different endosphere genera were observed, indicating that endophytic microbial diversity was relatively stable within the *P. sibiricum* rhizome, especially for endosphere bacteria. Cointertia analysis (CoIA) suggested that the metabolite changes in *P. sibiricum* induced by interplanting under different trees were more strongly affected by rhizosphere microorganisms than by endosphere microorganisms. In addition, the interactions between rhizosphere microorganisms and metabolites in the farmland group were weakened compared with those in the underplanted groups. Canonical correspondence analysis (CCA) showed that *Aspergillus* and *Ellin6067* had the greatest influence on the metabolites. *Myrmecridium*, as a shared microbe in the rhizosphere and endosphere, had interaction effects with the largest number of microbes.

**Conclusion:**

This study revealed the interactions between the microbes and metabolites in *P. sibiricum* and systematically explored the mechanism underlying their correlation, which was mediated by the understory interplanting mode. This study provides feasible strategies for improving the medicinal value of *P. sibiricum* by regulating microorganisms.

## 1. Introduction

*Polygonum sibiricum* Delar. ex Redoute, Lil (*P. sibiricum*), is a source of traditional herbal medicine in the lily family, an important understory economic plant in China, and a member of the Pharmacopoeia of the People’s Republic of China. As a traditional Chinese medicine (TCM), the dried rhizome of *P. sibiricum* contains rich polysaccharides and saponins and has high medicinal value due to its antiaging and immunity regulation effects.

The potency of medicinal plants is affected by several environmental factors, such as plant-mediated microbial groups, soil nutrients and climate. In other words, TCM microecology will influence the growth and development process of medicinal plants and thus have an important impact on TCM potency. Recent studies have shown that the soil microbiome is the main driving factor for plant growth. For example, rhizosphere or endosphere microorganisms can affect the growth and disease resistance of *Salvia miltiorrhiza*, *Panax notoginseng* and *Angelica sinensis*. Related beneficial microorganisms can promote nutrient absorption by plant roots (Zhang et al., 2020) and enhance plant resistance to diseases and insects (Nakaew et al., 2019). Salt resistance (Egamberdieva et al., 2016), drought resistance (Sedigheh et al., 2018) and heavy metal resistance were also improved by some related benefical microorganisms (Han et al., 2019). For example, mycorrhizal fungi increased the levels of nitrogen, phosphorus, potassium and other nutrient elements in the rhizosphere soil of *Paris polyphylla*, which increased nutrient element enrichment and was beneficial to quality formation (Morgan et al., 2005). Some rhizosphere microorganisms can also stimulate the production of plant hormones that promote growth and promote the synthesis of active components in medicinal plants. Studies have shown that *Burkholderia*, which is significantly enriched in the rhizosphere of *Radix isatidis*, is involved in the synthesis of indigo (Zhai et al., 2019). *Trichoderma asperellum* can increase the expression of genes related to artemisinin biosynthesis (Jiang et al., 2018). *Panobacterium* and *Enterobacterium* were found to be positively associated with polysaccharide and saponin levels, while *Pseudomonas* was significantly positively correlated with the total flavonoid level in *P. sibiricum* according to the community structure of endosphere microorganisms and active component levels (Cai et al., 2021).

Conversely, plants can also shape rhizosphere or endosphere microbial community structures. Plant root exudates, such as sugars and amino acids, can regulate the expression of genes related to chemotaxis, secondary metabolism and signal response in rhizosphere microorganisms and thus promote the colonization and growth of microorganisms in the rhizosphere (Peng et al., 2020). Studies have shown that aromatic hydrocarbon volatiles released by the *Atractylodes lancea* rhizome can change the rhizosphere fungal community and thus inhibit the growth of the pathogen *Fusarium* (Li et al., 2018). The triterpenoids produced by *Arabidopsis* roots can regulate the growth of specific bacteria in the *Arabidopsis* rhizosphere (Huang A. C. et al., 2019). At the same time, these plant-mediated microbial changes in soil can affect the growth of plant species that appear later in the same soil, and this effect is called plant–soil feedback (Hannula et al., 2021). Plants can negatively affect subsequent plant species through the accumulation of pathogenic microorganisms in the soil or positively affect them through the accumulation of beneficial microorganisms; however, this legacy effect is still poorly understood. Studies have shown that understory planting can provide a healthy microbial environment for the optimal growth of medicinal plants. The relative abundance of growth-promoting microorganisms in the rhizosphere of *P. notoginseng* planted under trees is higher than that in the rhizosphere of *P. notoginseng* in the field (Kui et al., 2021). Interestingly, compared with those planted under citrus trees, *Platycladus* trees, pine trees, peach trees and osmanthus trees, *P. sibiricum* individuals planted under oak trees exhibited better growth, less disease and higher yield (Chen et al., 2021). This may be because a thick humus layer formed more easily under the deciduous trees; this layer not only provides nutrients for *P. sibiricum* but also effectively maintains understory soil moisture. In addition, when planted under the same trees, the survival rate, yield and economic benefits of *Polygonatum kingianum* were closest to those of plants cultivated in the field, followed by those of plants planted under *Asparagus* fern and *Polygonum multiflorum* (Yan et al., 2016). The above studies further indicate the importance of selecting suitable tree species for interplanting medicinal plants when developing forest-medicine models. The role of plant-mediated microbial community structure changes in the quality of TCMs is worth exploring.

To date, several studies related to TCM microorganisms have mainly focused on continuous cropping obstacles, and important breakthroughs have been made for *P. notoginseng*, *ginseng* and *Rehmannia*. *P. sibiricum* is one of the genuine TCMs in Shandong Province, China, and the understory planting model has reduced the costs of labor, fertilizer and shading. Plant-mediated microbiome changes can affect plants subsequently growing in the same soil; however, the quality of understory-planted *P. sibiricum* as mediated by microorganisms has not been explored. This study will focus on the differences in rhizosphere and endosphere microorganisms between understory interplanting and field cultivation to explore the quality formation mechanism mediated by microorganisms. This study is expected to provide a scientific basis for the extraction of functional strains and biological fertilization strategies for improving the quality of *P. sibiricum*.

## 2. Materials and methods

### 2.1. Sampling sites and sample collection

*Polygonatum sibiricum* was planted in walnut (cultivar Xiangling), chestnut (cultivar Taiguo 5) and persimmon (cultivar Yangfeng) orchards at Xiancaogu Base, Tai’an City, Shandong Province, China (36°20′46″N, 116°96′20″E) in 2017, and field cultivation was used as a control. The climate of the test site is a continental subhumid monsoon climate. The average annual precipitation is 697 mm, the average annual temperature is 12.9° and the average annual sunshine duration is 2627.1 h. The soil was sandy soil, and the chemical composition of the soil was as follows: P, 132 mg/kg; N, 324 mg/kg; and K, 1024 mg/kg; with a pH of 6.87. To ensure that the soil factors at the four sites were as similar as possible during the experiment, the agricultural practices used, such as fertilization, irrigation and weeding, were consistent.

Healthy and uniform rhizomes of *P. sibiricum* were selected and planted in the above experimental sites in March 2020, and rhizomes and rhizosphere soil samples were collected in March 2022. Rhizosphere soil was collected according to methods described by Bulgarelli (Bulgarelli et al., 2015). For each collected rhizome, the excess soil was removed by vigorous shaking, and then the soil closely attached to the rhizome was rinsed away with phosphate-buffered saline (PBS) solution and collected. Subsequently, the rinsing solution for each of the 5 rhizomes in the same treatment was concentrated in a 50 mL centrifuge tube and repeated as a rhizosphere soil sample, with 3 replicates for each treatment. After the rhizosphere soil was collected, the rhizomes were placed into a centrifuge tube with sterilized PBS, washed repeatedly with continuous vortexing until clean, and then transferred to a new centrifuge tube for ultrasonic surface cleaning (Xiao et al., 2017a,b). The obtained rhizomes were frozen in liquid nitrogen and used for the detection of endophytic microorganisms. Another 12 rhizome samples were prepared for metabolite detection. Thus, a total of 36 samples (3 replicates × 4 understory planting modes; 12 rhizome samples for metabolites +12 rhizosphere soil samples for microorganisms +12 endosphere samples for microorganisms) were stored at −80°C until DNA was extracted.

### 2.2. DNA extraction and sequencing

A FastDNA SPIN Kit for Soil and DNA Secure Plant Kit were used to extract DNA from rhizosphere and endosphere microorganisms, respectively.

We used the primers F515 (5′ - GTGCCAGCMGCCGCGGTAA - 3′) and R907 (5′ CCGTCAATTCCTTTGAGTTT - 3′) to amplify the V4 + V5 region of 16S rRNA. The primers 2024F (5′- GCATCGA TGAAGAACGCAGC-3′) and 2409R (5′- TCCTCCGCTTA TTGATATGC-3′) were used to amplify the ITS2 gene. The PCR system volume was 30 μL in total, including 15 μL of PCR Mix (Thermo Fisher Scientific, Waltham, MA, USA), 0.2 μM primers and 10 ng of DNA template. The PCR procedure was as follows: 98°C for 1 min; 30 cycles of 98°C for 10 s, 50°C for 30 s, and 72°C for 30 s; and 72°C for 5 min. PCR products were detected by 2% agarose gel electrophoresis, followed by DNA purification using the GeneJET Gel Extraction Kit (Thermo Fisher Scientific, Waltham, MA, USA). The quality of the DNA library was evaluated by an Agilent Bioanalyzer 2100 system. Sequencing was performed using the Illumina HiSeq 2500 sequencing platform.

### 2.3. Metabolite extraction and LC–MS detection

The frozen rhizomes were quickly ground to powder in liquid nitrogen, and then 5 mL of precooled solution (acetonitrile:isopropyl alcohol:water at a ratio of 3:3:2) was added to 100 mg of powder. The solution was centrifuged at 12,000× *g* and 4°C for 10 min, and the supernatant was concentrated with rotary vacuum drying. Then, 0.6 mL of 70% methanol was added to dissolve the substance. The resulting solution was centrifuged, and the supernatant was absorbed and filtered through a 0.22 μm microporous filter membrane into a sample collection bottle. Ultra-performance liquid chromatography–tandem mass spectrometry (UPLC–MS) analysis was used to obtain metabolome data. The metabolites were identified based on the MWDB and multiple reaction monitoring (MRM) model of two-stage spectrum and triple quadrupole mass spectrometry (Xiao et al., 2017a,b).

### 2.4. Data analysis

MetaboAnalyst 2.0 was used for principal component analysis (PCA) of identified metabolites with Pearson correlation coefficients set to default values. Variable Importance in the Projection (VIP) > 1.0, fold change (FC) > 1.2 and *p* value <0.05 were set as thresholds to screen differentially abundant metabolites. Hierarchical clustering analysis of differentially abundant metabolites was performed using MultiExperiment Viewer-Tm4 (MeV) software. Kyoto Encyclopedia of Genes and Genomes (KEGG) enrichment analysis of differentially abundant metabolites was performed using the Nohe Source Data Analysis Platform, and the threshold was set as a *p* value ≤0.05.

Quantitative Insights Into Microbial Ecology (QIIME) was used to filter the original microbial reads. The remaining reads of the original DNA fragments were merged using the FLASH tool. Paired-end reads were matched to each sample based on QIIME unique barcodes. Operational taxonomic unit (OTU) identification was performed with the sequence similarity threshold set at 97%, and the RDP classifier was used to annotate representative sequences of each OTU. The alpha-and beta-diversity of the obtained OTUs were analyzed using Perl scripts. PCA based on weighted UniFrac (WUF) distances was performed using the “ape 3.4” package in R software. Permutational multivariate analysis of variance (PERMANOVA) was carried out using “vegan 2.3–0.” Canonical correspondence analyses (CCAs) were conducted using ade 1.7–4. Cointertia analysis (CoIA) was performed on the Tutools platform,^1^ a free online data analysis website. The correlation coefficients between differentially abundant microorganisms and metabolites were obtained using R software, and the coexpression network was visualized using Cytoscape software.

## 3. Results

### 3.1. Comparative analysis of metabolites in *P. sibiricum* interplanted under different trees

**Figure 1:**
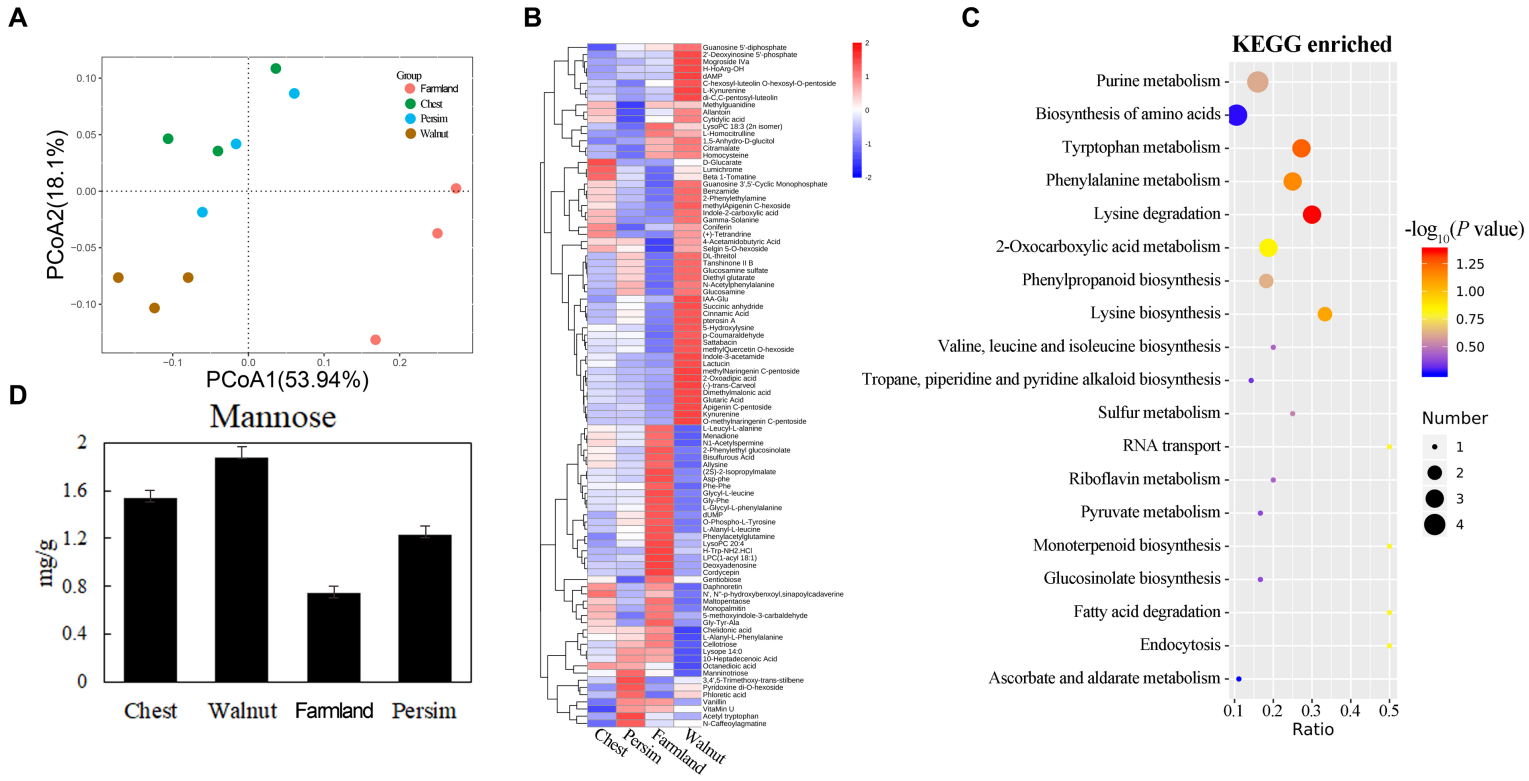
Effects of understory interplanting on secondary metabolism in the *P. sibiricum* rhizome. **(A)** Principal component analysis for metabolism of all of the groups. **(B)** Clustering analysis of the 95 differentially abundant metabolites. **(C)** Enrichment analysis for the 95 differentially abundant metabolites. **(D)** Mannose content in the four groups.

The PCA of metabolism (Figure 1A) showed significant differences in metabolic profiles among different groups (*p* < 0.05), and 12 samples could be significantly divided into 3 categories. The field cultivation group and the other three groups were significantly different in the axis 1 direction. The chestnut-interplanted groups and persimmon-interplanted groups were clustered together, indicating their similarity. A total of 862 metabolites were identified (Supplementary Table S1) in the 12 samples. Supplementary Table S1 shows that the differentially abundant metabolites in the chestnut group vs. field cultivation, walnut group vs. field cultivation, persimmon group vs. field cultivation, chestnut group vs. walnut group, chestnut group vs. persimmon group and walnut group vs. persimmon group comparisons numbered 14, 58, 14, 9, 5, and 17, respectively. A total of 95 differentially abundant metabolites (Supplementary Table S1) were observed in the 6 comparisons. We found that the number of differentially abundant metabolites between the walnut group and field cultivation was the largest and that between the chestnut group and walnut group was the smallest (Supplementary Table S1). Hierarchical clustering analysis (HCA) was conducted for the 95 differentially abundant metabolites obtained. Figure 1B shows that the 95 differentially abundant metabolites could be divided into 4 groups based on their expression patterns. Pattern 1 characterizes 53 metabolites that were highly expressed in *P. kingianum* rhizomes planted under walnut trees. Pattern 2 described 22 metabolites that were highly expressed in the *P. kingianum* rhizome in the field cultivation group. Pattern 3 characterized 12 metabolites, which were least expressed in the walnut group. Pattern 4 represented only 8 metabolites, which were highly expressed in the persimmon group. Pathway analysis (Figure 1C) showed that the 95 differentially abundant metabolites were mainly involved in signaling pathways such as purine metabolism, amino acid synthesis, tryptophan metabolism, phenylalanine metabolism, lysine degradation, phenylpropyl synthesis, and tryptophan synthesis. Polysaccharides were the main effective component in *P. sibiricum*, but the polysaccharide level was not significantly different among the groups. The polysaccharide components included mannose, galactose, arabinose and glucose (Wang, 2020). Figure 1D shows that the mannose content significantly differed among the groups, and it was the lowest in the field cultivation group and highest in the walnut group. Mannotriose is involved in galactose metabolism, and its decomposition yields galactose, which is an important link in the galactose metabolism pathway. Figure 1B and Supplementary Table S1 show that mannotriose was highly expressed in the persimmon group. The above results showed that interplanting mode had significant effects on the metabolites in the *P. sibiricum* rhizome.

### 3.2. Effects of interplanting mode on the rhizosphere and endosphere microorganisms of *P. sibiricum*

A total of 3,267,438 high-quality reads were obtained from 48 samples, with an average of 68,071 reads per sample. The sequence similarity threshold was set to 97%. A total of 9,672 bacterial OTUs and 2,204 fungal OTUs were obtained from the rhizosphere soil. A total of 1812 bacterial OTUs and 995 fungal OTUs were obtained for endosphere microorganisms (Supplementary Table S2).

#### 3.2.1. Variations in microbial community alpha diversity

**Figure 2:**
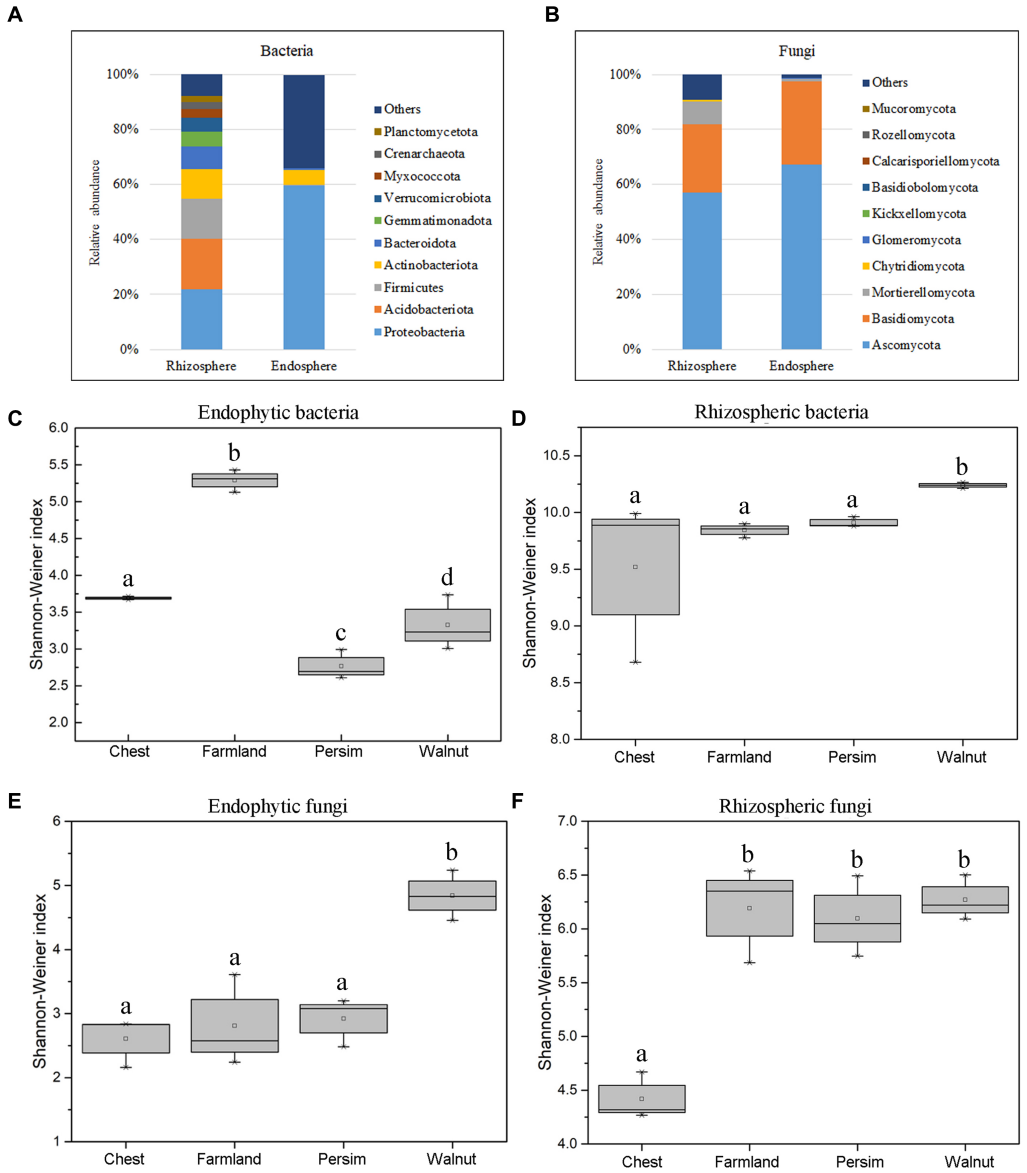
The microbial community colonizing the rhizosphere and root endosphere. **(A)** The relative abundance of bacteria at the phylum level. **(B)** The relative abundance of fungi at the phylum level. **(C–F)** Boxplot of the Shannon–Wiener index of the microbiome colonizing the rhizosphere and root endosphere.

According to Figures 2A,B, *Proteobacteria*, *Acidobacteria*, *Firmicutes* and *Actinobacteria* were the most abundant bacterial phyla in rhizosphere soil, accounting for 92.1% of the total bacteria. *Ascomycota, Basidiomycota* and *Mortierellomycota* were the most abundant fungal phyla, accounting for 90.2% of the total fungi. Among endosphere microorganisms, *Proteobacteria* and *Actinobacteria* were the most abundant bacterial phyla, and *Ascomycota* and *Basidiomycota* were the most abundant fungal phyla. As shown in Supplementary Figure S4, the top 10 dominant genera shared by the farmland and understory interplanting groups included *Tausonia*, *Solicoccozyma*, *Pseudomonas, Mortierella*, *Humicola* and *Fusarium. Gibberella* and *RB41* were the unique dominant genera in the field cultivation group, while *Talaromyces, Rhizobium* and *Setophoma* were the unique dominant genera in the understory interplanting groups. Alpha diversity analysis (Figures 2C–F) showed that for the endosphere, the bacterial Shannon–Wiener index in the field cultivation group was the highest, and the fungal Shannon–Wiener index in the walnut interplanting group was the highest. In the rhizosphere soil, the bacterial Shannon–Wiener index in the walnut interplanting group was the highest, and the fungal Shannon–Wiener index in the farmland group was the highest. The Kruskal–Wallis test was conducted on the Shannon–Wiener indexes of different groups, showing that the *p* values for endophytic bacteria, endophytic fungi, rhizosphere bacteria and rhizosphere fungi were 0.036, 0.046, 0.042, and 0.045, respectively. These results indicated that interplanting mode had significant effects on the alpha diversity of rhizosphere soil microorganisms and endophytic microbial community structure.

#### 3.2.2. Microbial community variation among different understory planting groups

**Figure 3:**
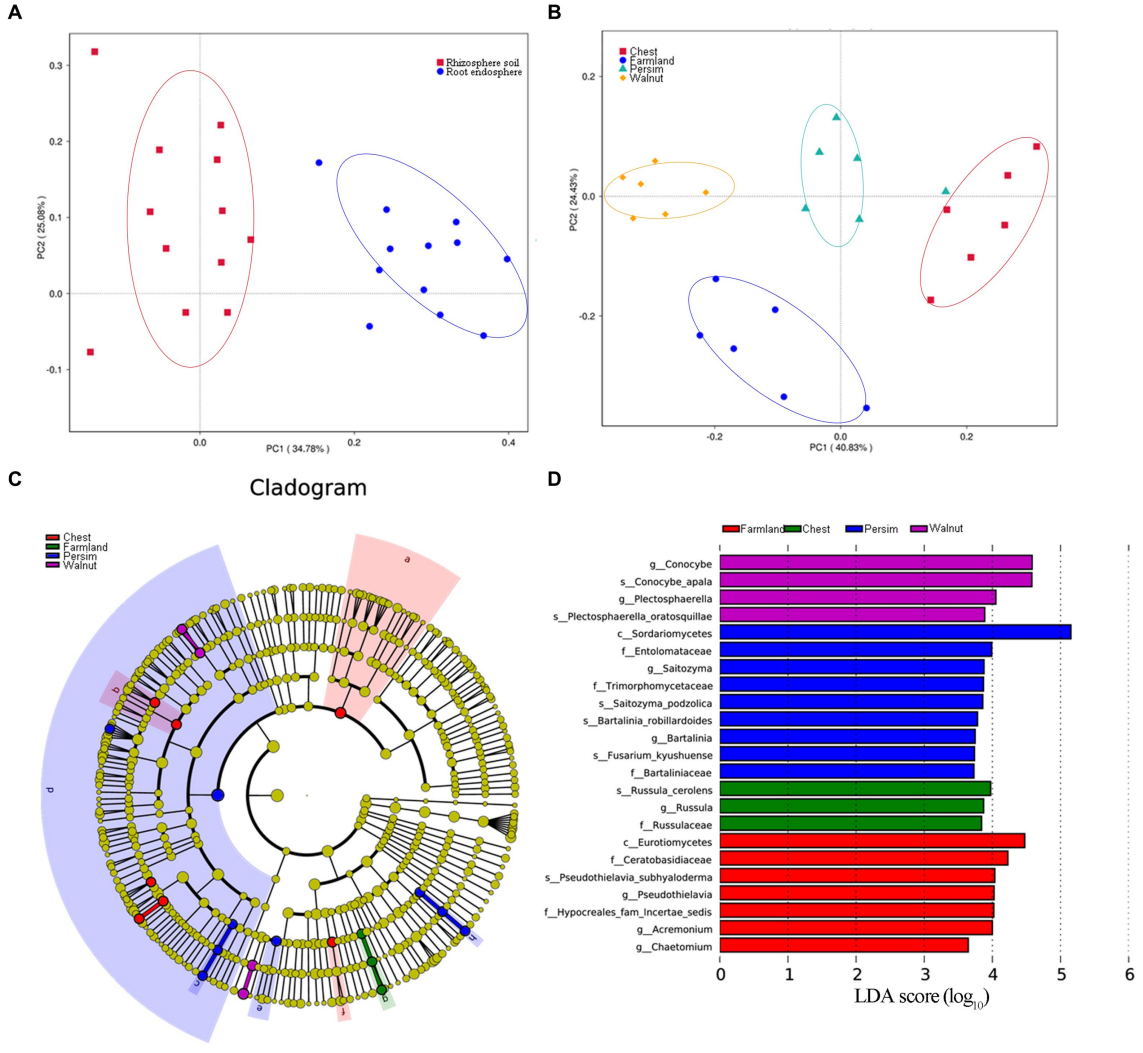
Beta analysis of the microbial community. **(A)** Principal component analysis for the microbiome grouped by rhizosphere and root endosphere. **(B)** Principal component analysis for the microbiome grouped by four interplanting groups. **(C,D)** Linear discriminant analysis effect size (LEfSe) results of rhizospheric fungi for four interplanting groups, illustrating a total of 23 characteristic fungal genera.

Principal component analysis (Figures 3A,B) showed that the microbial community structures of the rhizosphere and endosphere were significantly different and obviously divided into two classes. The microbial communities of *P. sibiricum* with different understory interplanting modes were clearly divided into four categories, among which the farmland group and three understory interplanting groups were significantly separated on axis 2. The chestnut and persimmon groups were closest to each other, indicating that the microbial community structure in the two groups was similar, while the chestnut and walnut groups were farther apart, indicating that the microbial community structure in the two groups was the most different. PERMANOVA was performed to further analyze how the two grouping factors (different tissues and different understory interplanting modes) explained differences in microbial structure. As shown in Supplementary Table S3, the contributions of understory interplanting mode to rhizosphere bacteria, rhizosphere fungi, endophytic bacteria and endophytic fungi were 50.88% (*p* = 0.006), 48.52% (*p* = 0.006), 13.47% (*p* = 0.057) and 31.06% (*p* = 0.041), respectively. The contributions of sampled tissue to bacteria and fungi were 52.06% (*p* = 0.006) and 11.42% (*p* = 0.071), respectively. These results indicated that both sampled tissue and understory interplanting mode had significant effects on microbial structure, and the influence of understory interplanting mode on beta diversity was greater in the rhizosphere than in the endosphere.

The linear discriminant analysis (LDA) effect size (LEfSe) technique was applied to explore the microorganisms with distinctive identifying properties for each group. The LDA threshold was set as 3.5, and an all-against-all analysis strategy was adopted. According to Figures 3C,D, a total of 23 identified rhizosphere fungal genera were found in the 4 groups. *Conocybe* and *Plectosphaerella* were the identifying fungal genera of the walnut group, *Saitozyma* and *Bartalinia* were the identifying fungal genera of the persimmon group, *Russula* was the identifying fungal genus of the chestnut group, and *Pseudothielavia*, *Acremonium* and *Chaetomium* were the marker fungal genera in the field cultivation group. A total of 20 identified rhizosphere bacterial genera were found in the four groups (Supplementary Figures S1A,B); among them, the farmland group had the largest number of marker bacterial genera, and the relative abundance of *Acidobacteria* was the highest, while there were no observed marker bacterial genera in the chestnut group. According to Supplementary Figures S1C,D, 12 marker endophytic fungal genera were identified in the four groups. *Volutella, Rhizopus, Knufia* and *Edenia* were identified as characteristic endophytic fungal genera in the walnut group, persimmon group, chestnut group, and field cultivation group, respectively. In contrast to the results for the endophytic fungi, no statistically characteristic endophytic bacterial genera were found in the four groups.

To further investigate the microorganismal differences between groups, Metastat analysis was performed, and the results are shown in Supplementary Tables S4–S7. In the farmland vs. permission group comparison, there were 38 genera of differentially expressed fungi (DEFs) and 52 genera of differentially expressed bacteria (DEBs) in the rhizosphere and 5 DEF genera and 0 DEB genera in the endosphere. In the farmland vs. walnut group comparison, there were 35 DEF genera and 124 DEB genera in the rhizosphere and 8 DEF genera and 0 DEB genera in the endosphere. In the chestnut vs. farmland group comparison, there were 32 DEF genera and 75 DEB genera in the rhizosphere and 4 DEF genera and 0 DEB genera in the endosphere. In the chestnut vs. permission group comparison, there were 21 DEF genera and 36 DEB genera in the rhizosphere and 2 DEF genera and 0 DEB genera in the rhizosphere. In the chestnut vs. walnut group comparison, there were 17 DEF genera and 53 DEB genera in the rhizosphere and 4 DEF genera and 10 DEB genera in the endosphere. In the walnut vs. persimmon group comparison, there were 27 DEF genera and 83 DEB genera in the rhizosphere and 8 DEF genera and 0 DEB genera in the endosphere. In conclusion, 217 rhizosphere DEB genera, 59 rhizosphere DEF genera, 23 endosphere DEF genera and 10 endosphere DEB genera were identified by pairwise comparison via Metastat analysis.

#### 3.2.3. Correlation between rhizosphere and endosphere microorganisms in *P. kingianum*

Supplementary Figure S2 shows that 725 bacterial genera were observed in the rhizosphere soil, and 290 bacterial genera were annotated in the endosphere. A total of 497 bacterial genera were expressed uniquely in the rhizosphere soil and 62 in the endosphere, while 228 shared bacterial genera were observed, accounting for approximately 78.6% of the total endophytic bacterial genera. A total of 327 fungal genera were annotated in the rhizosphere soil, and 212 fungal genera were annotated in the endosphere. In total, 180 rhizosphere fungal genera and 65 endosphere fungal genera were expressed uniquely; meanwhile, 147 shared fungal genera, accounting for 69.3% of the total endophytic fungal genera, were observed. Among the 228 shared bacterial genera, 7 genera, including *Pseudomonas, Chloroplast, Burkholderia, Ralstonia, Cupriavidus* and *Nocardia*, had a relative abundance of more than 1% in the *P. sibiricum* rhizome. Eight genera, including *Rb41, Sphingomonas, Gemmatimonas, Bacteroides* and *Bryobacter*, had a relative abundance greater than 1% in rhizosphere soil. Among the 147 shared fungal genera, 8 genera, including *Fusarium, Humicola, Mortierella, Solicoccozyma* and *Gibberella*, had a relative abundance greater than 1% in the *P. sibiricum* rhizome. *Fusicolla, Penicillium, Mycochlamys, Trichoderma, Botryotrichum* and 9 other genera had relative abundances greater than 1% in rhizosphere soils.

#### 3.2.4. Interactions between differential microbes and metabolites

**Figure 4:**
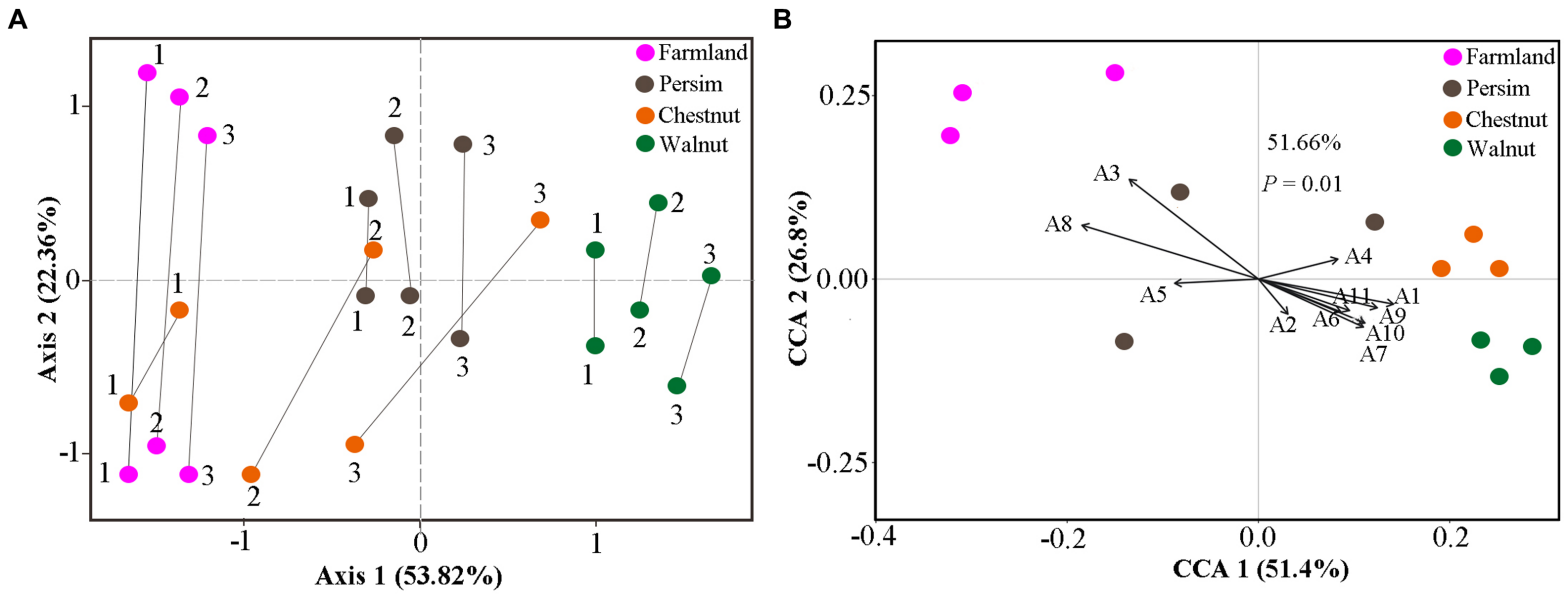
Cointertia analysis (CIA) and canonical correlation analysis (CCA) for rhizospheric samples in the farmland, walnut, persimmon and chestnut groups. **(A)** CIA results illustrated the covariation between metabolites and rhizospheric bacteria. Each group contains six nodes, three of which represent the metabolome, and the other three nodes represent the microbiome. The more parallel the line segments are, the stronger the covariation between the metabolome and microbiome. **(B)** CCA results illustrated the effects of typical differentially expressed microbes on metabolic changes. A1–A11 represent *Mortierella, Tausonia, Aspergillus, RB41, Candidatus_Solibacter Blautia, Bacillus, Ellin6067, Ruminococcus_gnavus, MND1* and *Terrimonas*.

To explore whether the changes in differentially abundant metabolites are related to differentially expressed microbes, we used CIA analysis to determine the covariation in 95 differentially abundant metabolites and 217 rhizosphere DEB genera, 59 rhizosphere DEF genera, 23 endosphere DEF genera, and 10 endosphere DEB genera. As shown in Figure 4A and Supplementary Figure S3A, multiple lines between metabolites and microbes in rhizospheric samples were parallel overall, indicating that the covariation of metabolites and microbes were similar. The shortest line segment in the walnut group indicated that the interaction between rhizosphere microbes and metabolites was the strongest in the rhizome of *P. sibiricum* interplanted under walnut. The longest line segment in the farmland group indicated that the interaction between rhizosphere microbes and metabolites was the weakest in field-cultivated *P. sibiricum*. Compared with the results for the rhizosphere, the parallel state of lines between endosphere microbes and metabolites was less obvious (Supplementary Figure S3B), indicating that the covariation between endophytic microbes and metabolites was weak. These results suggested that the metabolite changes were more strongly affected by rhizosphere microorganisms than by endosphere microorganisms.

Canonical correspondence analysis was conducted to explore the specific effects of differentially abundant microbes on metabolites in *P. sibiricum* for 95 metabolites and 11 typical differentially expressed rhizosphere microbes (Supplementary Table S8). The results in Figure 4B show that 11 differentially expressed genera could explain 51.66% of the metabolite variation. Ninety-five differentially abundant metabolites were separated in the direction of *Mortierella*, *Ellin6067*, and *Ruminococcus_gnavus*. The lines representing *Aspergillus* and *Ellin6067* were the longest, indicating that they had the greatest influence on the metabolite variations in *P. sibiricum*. In addition, Figure 4B shows that the farmland group was closest to *Aspergillus*, the walnut group was closest to *Mortierella* and the chestnut group was closest to *RB41*, suggesting that *Aspergillus, Mortierella* and *RB41* had the strongest effects on the metabolite differences of the farmland group, walnut group and chestnut group, respectively.

**Figure 5:**
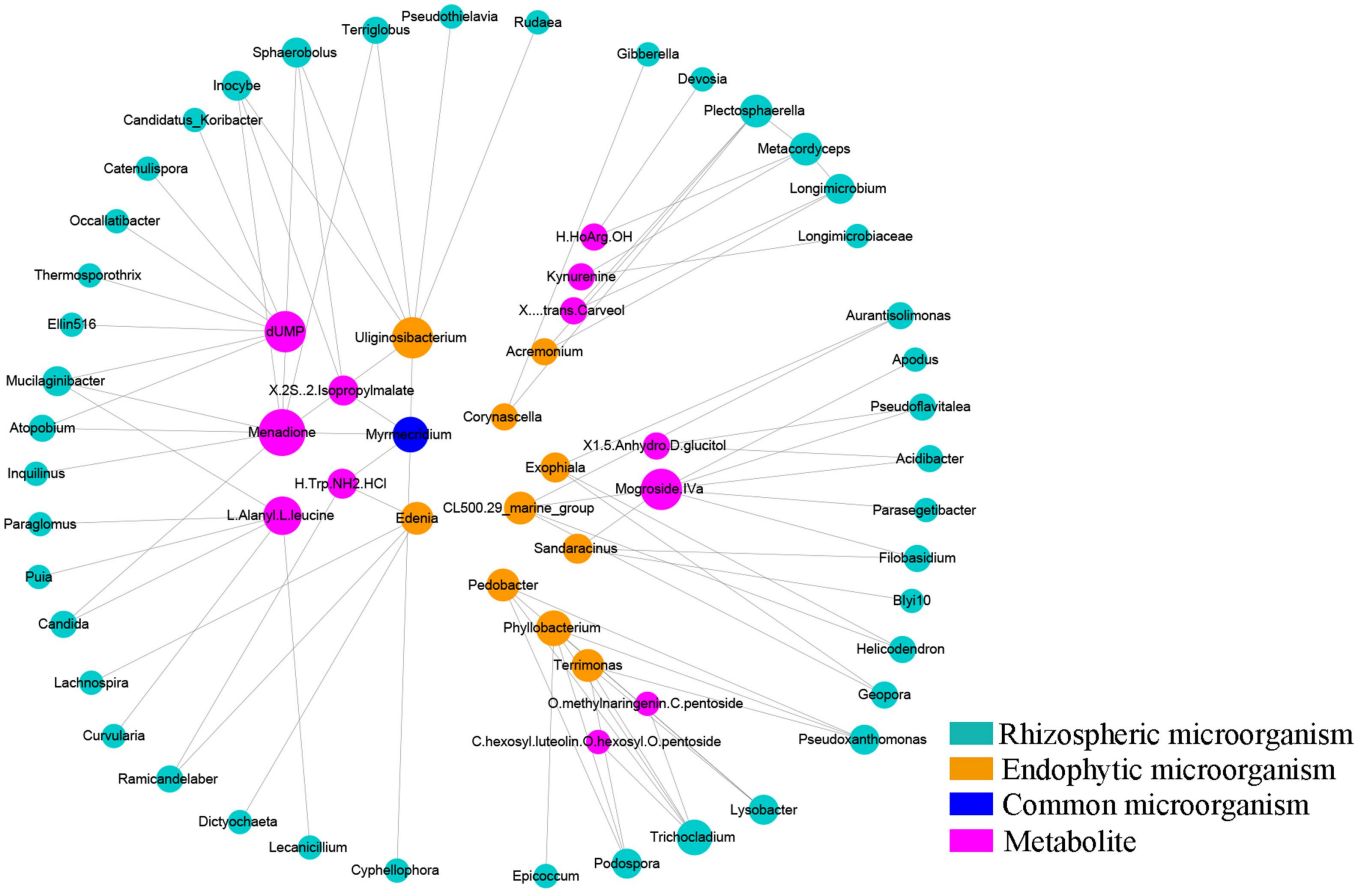
Coexpression analysis between differentially abundant microbes and metabolites. Cycle nodes represent microbes or metabolites. The size and color of nodes represent the degree of interactions among them. The larger the node is, the greater the number of nodes that interact with it.

Coexpression analysis was performed for 95 metabolites, 272 differentially expressed rhizosphere genera and 33 differentially expressed endosphere genera obtained by Metastat analysis. To ensure strong interactions, relationships with correlation coefficients greater than 0.9 or less than-0.9 were used to construct the coexpression network. The coexpression network contained 113 pairs of correlations and 102 nodes, including 53 rhizosphere microbes, 21 endosphere microbes and 23 metabolites (Supplementary Table S9). Figure 5 shows the core of the coexpression network. According to Figure 5 and Supplementary Table S9, four metabolites, namely, menadione, dUMP, mogroside Iva and L-alanyl-L-leucine, were at the core of the coexpression network. The number of microbes interacting with these four metabolites was 9, 7, 7 and 6, respectively. Among the root microorganisms, *Uliginosibacterium* was the genus with the most interaction effects, showing interactions with 7 kinds of rhizosphere microbes. In addition, we found that *Myrmecridium*, as a common microbe of the rhizosphere and endosphere, interacted with several metabolites, such as Cyphellophora, Uliginosibacterium, Menadione and (2S)-2-isopropylmalate, and had a high degree of interaction in this coexpression network. These results indicated that Myrmecridium plays an important role in the network composed of rhizospheric microbes, endophyte microbes, and metabolites, which is influenced by understory interplanting.

## 4. Discussion

### 4.1. Effects of interplanting tree type on the growth and development of *P. sibiricum*

Understory intercropping can provide shade conditions for *P. sibiricum* and prevent the damage caused by high summer temperatures, and the physical and chemical properties of soil could also be improved (Yong et al., 2019); therefore, tree type is one of the main factors affecting *P. sibiricum* growth. In this study, the mannose content of *P. sibiricum* in the interplanting groups was significantly higher than that in the farmland group, and the mannose content in the walnut group was the highest. It has been reported that some plant growth-promoting rhizobacteria, such as *Acinetobacter* and *Streptomyces*, increased the concentrations of mannose, glucose and galactose in maize (Lopes et al., 2022); meanwhile, the relative abundance of *Verrucomicrobiae* and *Proteobacteria* was strongly associated with a change in the proportion of mannose in *Acropora muricata*, *Vibrio coralliilyticus* and *Vibrio natriegens*, which may potentially be able to utilize a large amount of mannose (Sonny et al., 2016). In our study, the increase in mannose content in *P. sibiricum* planted under walnut may be related to specific bacteria.

In addition, compared to differences within the three interplanting groups, the number of different metabolites between the farmland group and the three groups was larger (Supplementary Table S1). Similarly, *P. sibiricum* planted under oak trees had better growth, less disease and higher yield than that planted under citrus trees, *Platycladus* trees, pine trees, peach trees and laurel trees (Chen et al., 2021). This may be related to ecological factors such as scattered light, air temperature and air humidity (Huang et al., 2016) or differences in rhizosphere microorganisms caused by tree interplanting (Fang et al., 2022). Specifically, rhizospheric microorganisms could affect the nutrient availability, resistance, and antagonism of pathogenic microorganisms (Zhu et al., 2021a,b). Habitat conditions are one of the factors that affect the growth and accumulation of secondary metabolites in *P. sibiricum*. In this study, 53 secondary metabolites were highly expressed in the walnut group, possibly because shading under walnut trees is more suitable for *P. sibiricum* growth because excessive shading or no shading could inhibit growth (Ni et al., 2020). Although there were significant differences in secondary metabolism among the groups, no significant differences were observed in morphology or polysaccharide content, which may be because the experimental period was only 2 years and the accumulation effect had not yet occurred.

### 4.2. Differences in microbial community diversity and composition in *P. sibiricum*

In this study, the metabolome and microbiome were analyzed for different understory interplanting groups. The PCA of the metabolome and microbiome separated the farmland group and understory interplanting groups along axis 1 and axis 2, respectively, suggesting differences in metabolism and microorganisms between the farmland group and understory interplanting groups. Similarly, in a previous study, the bacterial structure of *ginseng* in the understory interplanting group was more similar to that in the wild group than that in the farmland cultivation group (Fang et al., 2022). Compared with that in the farmland cultivation group, the alpha diversity of rhizosphere bacteria and endosphere fungi in the three understory interplanting groups was higher; however, the alpha diversity of rhizosphere fungi and endosphere bacteria was significantly decreased in the three understory interplanting groups. A series of farmland management measures, such as artificial shade and fertilization, may have affected the soil characteristics experienced by the farmland cultivation group (Huang, 2019), which then affected the intrinsic characteristics of *P. sibiricum*. In addition, *P. sibiricum* is susceptible to diseases and pests in the process of artificial cultivation of rhizomes in farmland (Rahman and Punja, 2006); therefore, pesticide spraying can indirectly affect rhizosphere secretions or directly inhibit the propagation of some rhizosphere microorganisms (Arango et al., 2014; Chen et al., 2017), thus affecting rhizosphere microbial diversity. Cultivated crops have the characteristics of fast growth and high yield, which may have led to differences in the amount and type of organic compounds secreted by the *P. sibiricum* rhizome and thus differences in rhizosphere microorganisms between the farmland group and the understory interplanting groups.

The diversity of rhizosphere fungi and bacteria showed opposite differences between the farmland cultivation group and understory interplanting groups; that is, the alpha diversity of rhizosphere bacteria (Shannon–Wiener index) significantly increased while that of rhizosphere fungi decreased in the understory interplanting group. A similar phenomenon has also been found in soybean studies (Shi et al., 2019). Modern selective breeding of crops may promote the propagation of certain crop-associated microorganisms, leading to changes in the diversity of fungi in cultivated crops (Wissuwa et al., 2009). Second, previous studies have shown that *ginseng* in understory interplanting groups contains a higher content of saponins than that in farmland cultivation groups (Choi et al., 2007), which may affect the diversity of crop rhizosphere fungi (Zhang et al., 2016).

This study showed that *Proteobacteria, Acidobacteria* and *Pseudomonas* were the dominant bacteria in both the rhizosphere and endosphere of *P. sibiricum*, and *Acidobacteria* was one of the marker bacteria in the farmland cultivation group. Previous studies have also shown that *Proteobacteria, Acidobacteria* and *Pseudomonas* are dominant among the rhizosphere microorganisms of *ginseng* and *P. sibiricum* (Ying et al., 2012; Cai et al., 2021). In fungal communities, *Fusarium* is a potential plant pathogen that can cause root rot in a variety of plants, such as *ginseng*, American ginseng, soybean, and sunflower (Punja et al., 2007; Ying et al., 2012; Okello et al., 2020; Wei et al., 2020). In this study, Simper test analysis showed that the relative abundance of *Fusarium* in the *P. sibiricum* cultivated in farmland was significantly higher than that in *P. sibiricum* in the other three interplanting groups. Similarly, cultivated rice has a higher pathogen abundance than wild rice (Shi et al., 2019). Our results showed that the dominant microbial genera were different under different planting modes (Supplementary Figure S4), which should be closely related to planting vegetation. In fact, different species accumulate specific fungal pathogens in their roots, showing plant species-specific effects (Hannula et al., 2021). In our study, Talaromyces, Setophoma and Rhizobium were the unique dominant genera in the chestnut, persimmon and walnut interplanting groups; interestingly, mannose in root secretions can be used by 100% of Rhizobium species, thus attracting more Rhizobium (He et al., 2011). This is consistent with the highest mannose content being observed in the walnut interplanting group. A potential caveat of our study was that we did not measure soil chemical-mediated legacy effects, which have been shown to play a role in mediating plant legacy effects in some studies (Petermann et al., 2008). However, we tried to keep agronomic measures consistent so that there were no significant differences in soil chemistry.

### 4.3. Potential mechanisms of association between microorganisms and metabolites in *P. sibiricum*

The plant microbiome is closely related to the synthesis and accumulation of secondary metabolites in medicinal plants. The rhizosphere is an important site for plant–soil–microbe information and material exchange, and the interaction between plant roots and rhizosphere microorganisms is crucial for plant growth and quality. At the same time, many plant endophytes also have important biological value and establish special relationships with host plants. They play an important role in the formation of active metabolites in medicinal plants and then affect the quality and yield of medicinal materials. The metabolism of active components in different plants is affected differently by rhizosphere microorganisms and endophytic microorganisms (Zhou et al., 2022). This study showed that the differences in *P. sibiricum* metabolites were more strongly affected by rhizosphere microorganisms than by the endophytic microorganisms. In addition, LEfSe analysis and Metastat analysis showed that the differences in endophytic bacteria among the four groups were minimal, and the alpha diversity of rhizosphere microbes was higher than that of endophytic bacteria. The results suggested that the influence of interplanting under different trees on the rhizosphere microbiota was greater than that on the endophytic bacteria and the stability of plant endophytic bacteria (Edwards et al., 2015). The shared bacteria and fungi in the rhizosphere and endosphere accounted for 78.6 and 69.3% of the endophytic microorganisms, respectively, indicating that most of the microorganisms that did not colonize the surface of the rhizosphere also did not colonize the root (Edwards et al., 2015) since the barrier effect of the root surface selects for certain endophytic microorganisms (Zhou et al., 2022).

This study showed that *Mortierella*, *Aspergillus*, *Ellin6067* and *Ruminococcus* could significantly distinguish the differentiated metabolites mediated by understory interplanting. Meanwhile, CCA suggested that *Mortierella, Aspergillus* and *RB41* had the strongest influence on the metabolites in the walnut group, farmland group and chestnut group, respectively. *RB41* is thought to play an important role in the maintenance of plant rhizosphere soil ecology (Shi et al., 2021). In addition, coexpression network analysis showed that menadione, dUMP, mogroside and L-alanyl-L-leucine had the most interactions with microorganisms. At the same time, *Myrmecridium*, as a common microbe in the rhizosphere and endosphere, also had a high degree of interaction in this coexpression network. *Myrmecridium* is believed to be able to antagonize diseases caused by continuous cropping disorders by affecting secondary metabolism in various medicinal plants (Dong et al., 2016; Wu et al., 2021). However, *RB41* and *Myrmecridium* are rarely studied and still need further study. These results suggest that there is a potential mechanism of association between the rhizosphere or rhizosphere microorganisms and secondary metabolism.

Indeed, studies have shown that the microbiomes of several plants can synthesize secondary metabolites, some of which contribute to the quality of medicinal plants. Inoculation with endophytic bacteria isolated from alkaloid-rich rose significantly increased the malsaline content of low-yielding alkaloid-poor rose (Singh et al., 2020; Zhu et al., 2021a,b). Plant growth-promoting rhizobia (PGPRs) in *Platycodon* (Huang C. M. et al., 2019), mycorrhizal fungi in *ginseng* (Kim et al., 2017) and *Actinomycetes* in *turmeric* all significantly promote plant growth and resistance (Kim et al., 2017). Conversely, amino acids, organic acids, sugars, and other small molecules secreted by roots can also affect the growth of some soil microorganisms (Hartmann et al., 2009). Studies have shown that rhizosphere bacteria preferentially utilize aromatic organic acids secreted by plants (Zhalnina et al., 2018), salicylic acid can regulate the colonization of the rhizosphere by specific bacterial groups (Lebeis et al., 2015), and metabolites such as triterpene can regulate the growth of specific bacterial groups in the rhizosphere of *Arabidopsis thaliana* (Huang A. C. et al., 2019). Among medicinal plants, *Salvia miltiorrhiza* serves as a model medicinal plant, and the mechanism of interaction between microorganisms and secondary metabolism has also begun to be studied. *Salvia miltiorrhiza* may have a unique microbiome that is rich in functions related to secondary metabolism (Huang et al., 2018); for example, *Pseudomonas* and *Pantoea* promote the synthesis of salvia phenol acid by stimulating the production of abundant plant hormones (Zhalnina et al., 2018). Microbial interactions with tanshinone may affect tanshinone biomass and metabolic pathways, and the main functional microorganisms were *Pantoea, Pseudomonas, Enterobacter, Sphingomonas and Alternaria* (Chen et al., 2018). Conversely, the presence of some secondary metabolites in *S. miltiorrhiza*, such as tanshinine I and dihydrotanshinine I, also has a significant effect on the structure of the endophytic microorganisms. Moreover, the expression patterns of *ginseng* secondary metabolites and the rhizosphere microbial community were similar (Zhu et al., 2021a,b). *Anthracis* and *Penicillium* in the endosphere showed a significant negative correlation with the polysaccharide content, while *Fusarium* showed an extremely significant positive correlation with 5-HMF in *P. sibiricum* (Cai et al., 2021).

## 5. Conclusion

Principal component analysis of the metabolome divided the four groups into two classes, and a total of 95 differentially abundant metabolites were found in pairwise comparisons, with four expression patterns. The alpha diversity of rhizosphere fungi and rhizosphere bacteria in the understory interplanting groups was significantly lower than that in the farmland cultivation group, but the alpha diversity of rhizosphere bacteria and rhizosphere fungi was higher than that in the farmland cultivation group. The CoIA results suggested that the metabolite changes induced by different understory interplanting treatments were more strongly influenced by the rhizosphere microbiota than by the endophytic microbiota, and understory interplanting enhanced the interactions between the rhizosphere microbiota and secondary metabolism. The CCA results and coexpression analysis suggest that *Aspergillus* and *Myrmecridium* play an important role in regulating metabolism in *P. sibiricum*. In future research, we will conduct isolation and inoculation tests on the selected core microbes to verify its role.

## Data availability statement

The datasets presented in this study can be found in online repositories. The names of the repository/repositories and accession number(s) can be found at: https://www.ncbi.nlm.nih.gov/, PRJNA975730.

## Author contributions

YW: data curation, writing-original draft preparation, and conceptualization. JZ and GL: conceptualization and methodology. JS: investigation. YZ: software. JH: supervision. CM: reviewing, editing, and funding acquisition. All authors contributed to the article and approved the submitted version.

## Funding

This work was funded by the National Natural Science Foundation of China (82104344), Shandong Province Science and Technology enterprises Innovation Ability Improvement Project of Shandong Province (2022TSGC2062), Tai'an agricultural seed improvement project of Shandong Province (2022NYLZ03), Science and Technology Innovation Project of Shandong Academy of Agricultural Sciences (CXGC2023B03), National Key Research and Development Program of China (2021YFD1900900), and the Key R&D Plan of Shandong Province (2021CXGC010801 and 2021CXGC010804).

## Conflict of interest

QW was employed by Shandong Taishang Huangjing Biotechnology Co., Ltd.

The remaining authors declare that the research was conducted in the absence of any commercial or financial relationships that could be construed as a potential conflict of interest.

## Publisher’s note

All claims expressed in this article are solely those of the authors and do not necessarily represent those of their affiliated organizations, or those of the publisher, the editors and the reviewers. Any product that may be evaluated in this article, or claim that may be made by its manufacturer, is not guaranteed or endorsed by the publisher.

## Footnotes

^1^http://www.cloudtutu.com

## Supplementary material

SUPPLEMENTARY FIGURE S1 Linear discriminant analysis effect size (LEfSe) results of the microbiome for four interplanting groups. **(A)** LefSe results for the rhizospheric bacterial community. **(B)** LefSe results for the endophytic fungal community.

SUPPLEMENTARY FIGURE S2 Venn diagram showing the unique and shared genera (3% distance level) in the rhizosphere soil and roots of *P. sibiricum*.

SUPPLEMENTARY FIGURE S3 The coinertia analysis (CIA) results of metabolism and the microbiome in four interplanting groups. CIA results illustrated the covariation between metabolites and rhizosphere fungi **(A)** and endosphere bacteria **(B)**. Each group contains six nodes, three of which represent the metabolome, and the other three nodes represent the microbiome. The more parallel the line segments are, the stronger the covariation between the metabolome and microbiome.

SUPPLEMENTARY FIGURE S4 The top 10 dominant microbial genera in the rhizosphere soil and root endosphere.

SUPPLEMENTARY TABLE S1 Details regarding metabolism in all samples.

SUPPLEMENTARY TABLE S2 Microbial populations annotated at each phylogenetic level in rhizosphere soil and root soil.

SUPPLEMENTARY TABLE S3 PERMANOVA of the microbial community composition of all samples based on WUF distance.

SUPPLEMENTARY TABLES S4-S7 Details of the Metastat analysis results for rhizosphere fungi, rhizosphere bacteria, endosphere fungi and endosphere bacteria.

SUPPLEMENTARY TABLE S8 The 11 typical differentially expressed rhizosphere microbes used to perform CCA.
