# Supplementary figures and images for: Insights into the mechanisms of microbiome and metabolome changes mediated by understory interplanting mode in Polygonatum sibiricum
Source: Front Microbiol. 2023 Aug 10;17:1218595. doi: 10.3389/fmicb.2023.1232846 (PMC10449124; doi:10.3389/fmicb.2023.1232846)

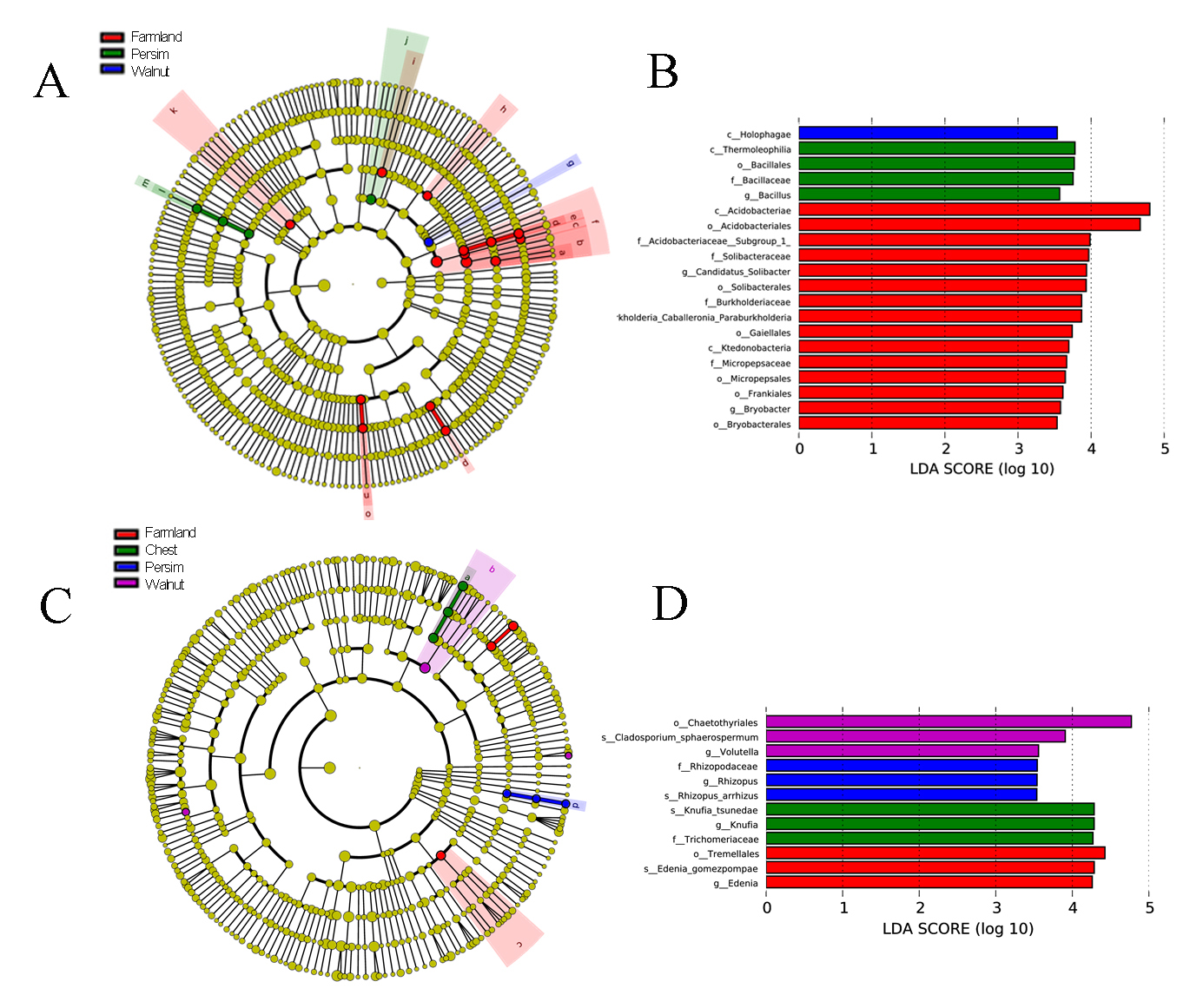

Supplement: SUPPLEMENTARY FIGURE S1 — Linear discriminant analysis effect size (LEfSe) results of the microbiome for four interplanting groups. (A) LefSe results for the rhizospheric bacterial community. (B) LefSe results for the endophytic fungal community. [file Image_1.JPEG]

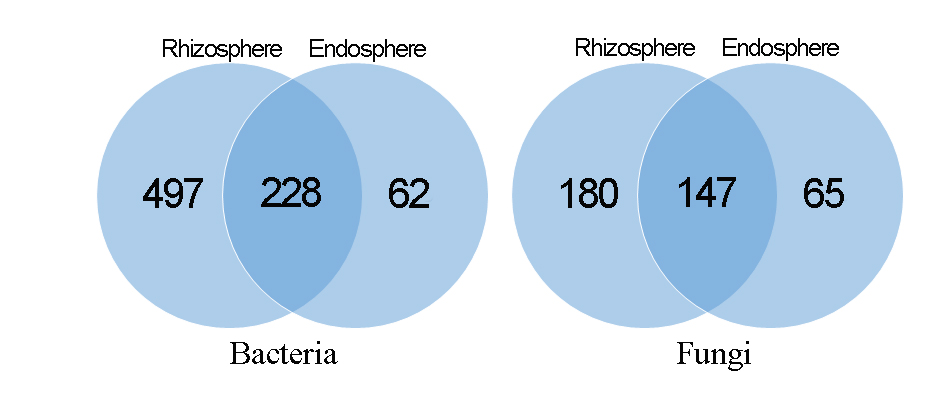

Supplement: SUPPLEMENTARY FIGURE S2 — Venn diagram showing the unique and shared genera (3% distance level) in the rhizosphere soil and roots of P. sibiricum. [file Image_2.JPEG]

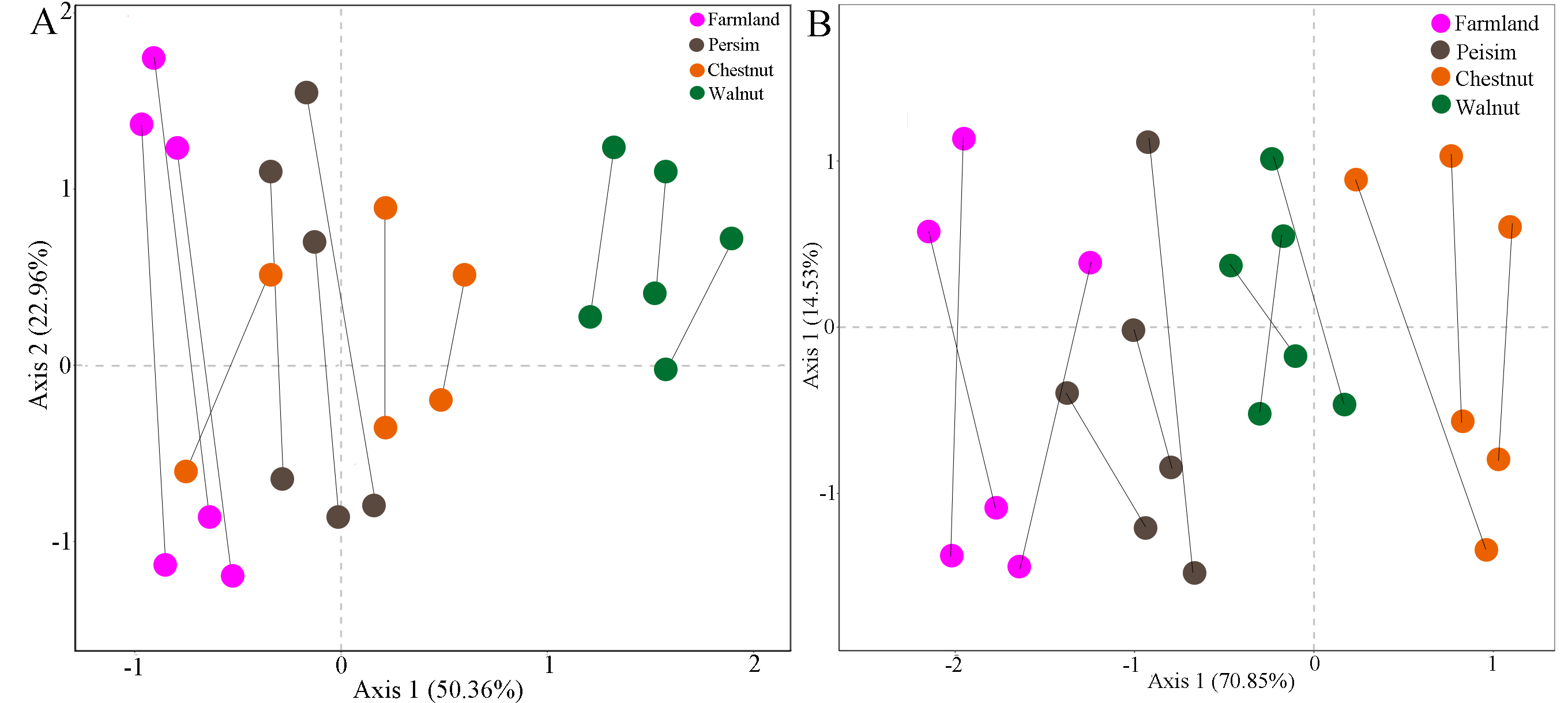

Supplement: SUPPLEMENTARY FIGURE S3 — The coinertia analysis (CIA) results of metabolism and the microbiome in four interplanting groups. CIA results illustrated the covariation between metabolites and rhizosphere fungi (A) and endosphere bacteria (B). Each group contains six nodes, three of which represent the metabolome, and the other three nodes represent the microbiome. The more parallel the line segments are, the stronger the covariation between the metabolome and microbiome. [file Image_3.JPEG]

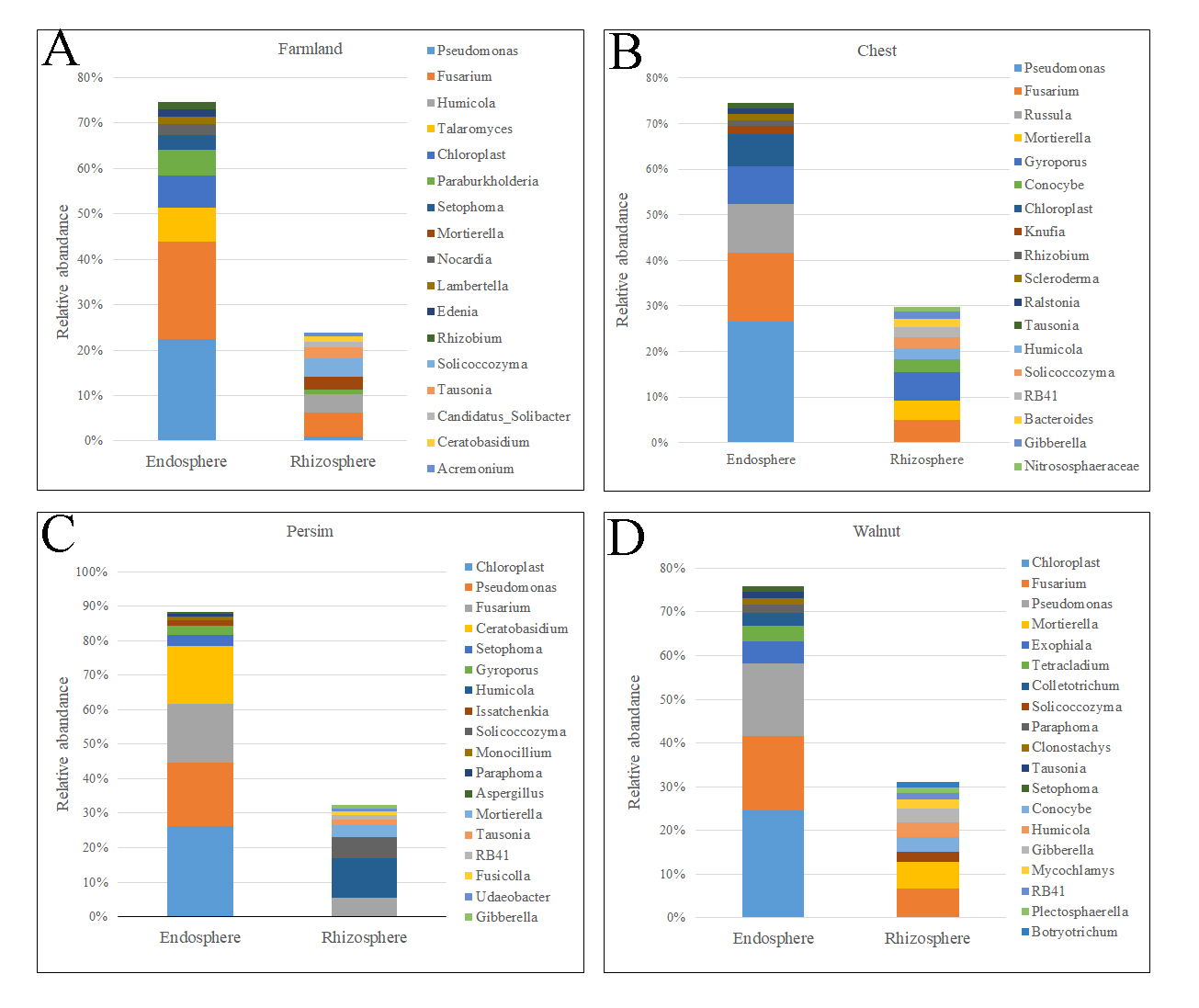

Supplement: SUPPLEMENTARY FIGURE S4 — The top 10 dominant microbial genera in the rhizosphere soil and root endosphere. [file Image_4.JPEG]
