# Supplementary material for: Insights into the mechanisms of microbiome and metabolome changes mediated by understory interplanting mode in Polygonatum sibiricum
Source: Front Microbiol. 2023 Aug 10;17:1218595. doi: 10.3389/fmicb.2023.1232846 (PMC10449124; doi:10.3389/fmicb.2023.1232846)
Supplement: SUPPLEMENTARY TABLE S2 — Microbial populations annotated at each phylogenetic level in rhizosphere soil and root soil. [file Table_2.DOCX]

Table S2 Microbial population that were annotated at each phylogenetic level in rhizosphere soil and root soil.

| **Taxa** | **Bacteria** | |  | **Fungus** | |
| --- | --- | --- | --- | --- | --- |
|  | **Rhizosphere soil** | **Root endosphere** |  | **Rhizosphere soil** | **Root endosphere** |
| OTU | 9672 | 1812 |  | 2204 | 995 |
| Phylum | 41 | 22 |  | 12 | 11 |
| Class | 101 | 39 |  | 32 | 25 |
| Order | 244 | 102 |  | 75 | 67 |
| Family | 393 | 176 |  | 175 | 138 |
| Genus | 724 | 288 |  | 326 | 212 |
