# Supplementary material for: Insights into the mechanisms of microbiome and metabolome changes mediated by understory interplanting mode in Polygonatum sibiricum
Source: Front Microbiol. 2023 Aug 10;17:1218595. doi: 10.3389/fmicb.2023.1232846 (PMC10449124; doi:10.3389/fmicb.2023.1232846)
Supplement: SUPPLEMENTARY TABLE S3 — PERMANOVA of the microbial community composition of all samples based on WUF distance. [file Table_3.DOCX]

|  | **Df** | **Sums Of Sqs** | **Mean Sqs** | **F. Model** | **R^2^** | **Pr(>F)** |  |
| --- | --- | --- | --- | --- | --- | --- | --- |
| **Bacteria in rhizosphere soil** | | | | | | | |
| Group | 3 | 0.3742 | 0.1247 | 1.1913 | 0.5088 | 0.006 | ** |
| Residuals | 8 | 0.8376 | 0.1047 |  | 0.4912 |  |  |
| Total | 11 | 1.2118 |  |  | 1 |  |  |
| **Fungi in rhizosphere soil** | | | | | | | |
| Group | 3 | 0.6638 | 0.2212 | 1.6709 | 0.4852 | 0.006 | ** |
| Residuals | 8 | 1.0593 | 0.1324 |  | 0.5148 |  |  |
| Total | 11 | 1.7231 |  |  | 1 |  |  |
| **Bacteria in root endosphere** | | | | | | | |
| Group | 3 | 0.6943 | 0.2314 | 1.3415 | 0.1347 | 0.03 |  |
| Residuals | 8 | 1.3800 | 0.1725 |  | 0.8653 |  |  |
| Total | 11 | 2.0743 |  |  | 1 |  |  |
| **Fungi in root endosphere** | | | | | | | |
| Group | 3 | 0.9226 | 0.3075 | 1.2019 | 0.3106 | 0.261 |  |
| Residuals | 8 | 2.0468 | 0.2558 |  | 0.6894 |  |  |
| Total | 11 | 2.9694 |  |  | 1 |  |  |

Table S3-1 PERMANOVA analysis of the microbial community composition of all samples grouped by four interplanting types based on WUF distance.

Table S3-2 PERMANOVA analysis of the microbial community composition of all samples grouped by rhizosphere or endosphere based on WUF distance.

|  | **Df** | **Sums Of Sqs** | **Mean Sqs** | **F. Model** | **R^2^** | **Pr(>F)** |  |
| --- | --- | --- | --- | --- | --- | --- | --- |
| **Bacteria** | | | | | | | |
| Compartment | 1 | 0.6457 | 0.1487 | 1.1853 | 0.5206 | 0.006 | ** |
| Residuals | 10 | 1.8539 | 0.2581 |  | 0.4794 |  |  |
| Total | 11 | 2.4996 |  |  | 1 |  |  |
| **Fungi** | | | | | | | |
| Compartment | 1 | 1.8573 | 0.4369 | 1.0231 | 0.1142 | 0.071 |  |
| Residuals | 10 | 2.6971 | 0.5126 |  | 0.8858 |  |  |
| Total | 11 | 4.5544 |  |  | 1 |  |  |
